# Haptoglobin expression correlates with tumor differentiation and five-year overall survival rate in hepatocellular carcinoma

**DOI:** 10.1371/journal.pone.0171269

**Published:** 2017-02-03

**Authors:** Chun-San Tai, Yan-Ren Lin, Tsung-Han Teng, Ping-Yi Lin, Siang-Jyun Tu, Chih-Hung Chou, Ya-Rong Huang, Wei-Chih Huang, Shun-Long Weng, Hsien-Da Huang, Yao-Li Chen, Wen Liang Chen

**Affiliations:** 1 Department of Biological Science and Technology, National Chiao Tung University, Hsinchu, Taiwan; 2 Institute of Molecular Medicine and Bioengineering, National Chiao Tung University, Hsinchu, Taiwan; 3 Program of Interdisciplinary Neuroscience, National Chiao Tung University, Hsinchu, Taiwan; 4 Department of Emergency Medicine, Changhua Christian Hospital, Changhua, Taiwan; 5 School of Medicine, Kaohsiung Medical University, Kaohsiung, Taiwan; 6 School of Medicine, Chung Shan Medical University, Taichung, Taiwan; 7 Transplant Medicine & Surgery Research Centre, Changhua Christian Hospital, Changhua, Taiwan; 8 Department of Medical Research, China Medical University Hospital, Taichung, Taiwan; 9 Institute of Bioinformatics and Systems Biology, National Chiao Tung University, Hsinchu, Taiwan; 10 Department of Medicine, Mackay Medical College, New Taipei City, Taiwan; 11 Department of Obstetrics and Gynecology, Hsinchu MacKay Memorial Hospital, Hsinchu, Taiwan; 12 Mackay Junior College of Medicine, Nursing and Management, Taipei, Taiwan; 13 Department of General Surgery, Changhua Christian Hospital, Changhua, Taiwan; University of North Carolina at Chapel Hill School of Medicine, UNITED STATES

## Abstract

Elevated serum haptoglobin (Hp) is identified as a prognostic marker in multiple types of solid tumors, which is correlated with poor prognosis. HCC is one of the major causes of cancer deaths in worldwide, which remains poor prognosis and is clinically urgent for discovering early diagnostic markers. However, except for serum Hp, the correlation of tumor Hp expression with hepatocellular carcinoma (HCC) progression is still unclear. In this study, we evaluated and identified the tissue Hp expression as a prognostic marker to predict the survival rate of HCC patients. To evaluate the prognostic value of Hp expression for HCC, two cohorts were enrolled in our study, including total 130 matched pair tissue sections (both adjacent non-tumorous and tumor tissue derived from same patient) of HCC patients from Changhua Christian Hospital (CCH) and total 316 RNA-seq data with clinical information of HCC patients from The Cancer Genome Atlas (TCGA) database. In contrast to other types of cancers, HCC tumor tissues have lower Hp protein expression in CCH cohort and have lower Hp mRNA expression in TCGA cohort as compared with adjacent non-tumorous tissues (*p* < 0.001). Moreover, lower Hp expression is significantly correlated with different stages of HCC cancer differentiation in CCH cohort (one-way ANOVA, *p* < 0.001). Most importantly, lower Hp expression is highly correlated with poor five-year overall survival rate in TCGA cohort (*p* < 0.01). Based on our data, we conclude that tissue Hp expression positively correlates with better HCC tumor differentiation and increased five-year overall survival rate of HCC patients. The results indicated that tissue Hp is potentially a prognostic marker for HCC patients. Our findings may further provide a new insight of effective treatments along with biopsy diagnosis of HCC patients.

## Introduction

Haptoglobin (Hp) is a secreted acute phase protein that is mainly produced in liver[1]. Elevated levels of Hp in plasma and locally in tumor tissue is closely associated with different clinical conditions, especially in various types of cancers such as ovarian cancer, colorectal cancer, pancreatic cancer, breast, and lung cancer[2–6]. Previous study reported the serum Hp could be regarded as an independent prognostic factor for epithelial ovarian cancer patients since patients with higher serum levels of Hp had significantly poor survival rates[7]. Additionally, Sun et al. demonstrated that serum Hp is highly expressed in colorectal cancer and is correlated with poor survival rate[8]. Moreover, it has been shown that the increased serum levels of Hp in non-small cell lung cancer (NSCLC) is associated with advanced TNM stages and distant metastasis[9]. These findings suggest the potential role of Hp in cancer prognosis and biomarker, which is worth and clinically urgent for further investigations.

Liver cancer is the second leading cause of cancer mortality and the fifth incidence rate among men and the ninth incidence rate in women[10]. Among all types of liver cancer, hepatocellular carcinoma (HCC) is the most commonly diagnosed type, representing 83% of all cases[11]. Currently, the critical issue for HCC is the poor five-year overall survival rate of only 30%-40%, which HCC patients often suffered postoperative recurrence and poor response to systemic chemotherapeutic treatments[12]. Despite the improvement in therapeutic treatments such as, radiotherapy, drugs, surgical resections or liver transplantation, the high incidence of tumor recurrence and metastasis still lead to poor prognosis of HCC patients[13]. Thus, identification of promising diagnostic markers as a direction for effective clinical treatments is worth for development. We here attempt to discover a prognostic marker in tissue level, which can be utilized with the biopsy for HCC diagnosis and, at the same time, provide more information for further therapeutic strategies to improve the overall survival rate of HCC patients. Unfortunately, besides the current pathological interpretation of tissue sections, there is still lack of promising tissue markers in predicting the outcome of HCC patients. Since the clinical significance of Hp is reported by previous studies and it is also highly expressed in liver, we hypothesized that Hp might possessed with a critically prognostic role in HCC development. Moreover, the differences of tissue Hp levels between tumor and adjacent non-tumorous parts (in the same patient) that may reflect the tendency of Hp secretion (under different cancer-related stress), which had never been well analyzed for prognostic value. Therefore, in this study, we aim to firstly identify tissue Hp level difference (between adjacent non-tumorous tissues and tumor tissues) as a prognostic marker for HCC outcomes using two cohorts, including prospective patients sample collections (n = 130) from Changhua Christian Hospital (CCH) and Hp mRNA analysis (n = 316) from The Cancer Genome Atlas (TCGA) database.

In this study, we enrolled a total number of 130 HCC patients from Changhua Christian Hospital (CCH) and 316 HCC patients from The Cancer Genome Atlas (TCGA) database. By using the immunohistochemistry staining for measuring the Hp protein expression of CCH tissue sections and computational methods analyzing for the Hp mRNA expression from TCGA datasets, we verified the tissue Hp expression between adjacent non-tumorous tissues and tumor tissues. We further analyzed the correlation of Hp expression with HCC cancer cell differentiation and five-year overall survival rate. Based on these data, we concluded that Hp serves as a prognostic marker for HCC patients. Higher tissue Hp expression is correlated with well-differentiated HCC cancer cells, which finally reflects on improved five-year overall survival rate.

## Materials and methods

### Changhua Christian Hospital (CCH) HCC patients

A total of 130 HCC patients, confirmed by histopathologic examination, were prospectively collected from Changhua Christian Hospital (Changhua, Taiwan) between 2011 to 2013. The study was approved by the institutional review board of the Changhua Christian hospital and written informed consent was obtained from all patients (IRB ID: 130704). The clinicopathological characteristics of enrolled HCC patients (gender, age at primary diagnosis, pathological stage, tumor size, pathological TNM classification, HCC cancer cell differentiation, hepatitis B history, liver cirrhosis history and alpha-fetoprotein) were shown in Table 1.

**Table 1:** Clinicopathological characteristics of CCH HCC patients.

	Patients with hepatocellular carcinoma (n = 130)		The percentage of Hp expression (Mean ± SD)			
	No.	%	In tumor part (%)	*p*-value	In adjacent non-tumorous part (%)	*p*-value
Gender						
Male	96	73.8	44.4±31.8	<0.01	90.7±10.6	0.22
Female	34	26.2	17.9±25.6		87.9±21.2	
Age (Mean ± SD) (y/o)	63.5 ± 10.3		-		-	
Pathologic_stage						
I	49	37.7	45.6±34.9	0.19	89.2±8.5	0.35
II	63	48.5	31.9±32.4		90.6±10.5	
III	16	12.3	35.5±31.5		91.6±16.4	
IV	2	1.5	30.0±14.1		77.5±31.8	
Tumor size (Mean ± SD) (mm)	56.9 ± 116		-		-	
Pathology_T_stage						
T1	47	36.2	45.9±34.8	0.18	89.4±8.6	0.79
T2	67	51.5	33.1±32.3		90.3±10.3	
T3	13	10	29.5±33.4		91.5±17.8	
T4	3	2.3	38.3±12.6		85.0±26.0	
Pathology_N_stage (19)*						
N-0	108	83.1	36.7±33.7	0.845	90.5±10.2	0.10
Non N-0	3	2.3	45.0±27.8		76.7±22.5	
Pathology_M_stage						
M-0	85	65.4	39.2±34.0	0.43	89.4±9.3	0.44
Non M-0	45	34.6	34.2±32.4		91.0±13.8	
HCC differentiation						
Well / Moderate	67	51.5	42.4±35.5	0.08	89.4±10.4	0.55
Poor	63	48.5	32.2±30.4		90.6±11.7	
Hepatitis B history						
Yes	78	60	38.2±33.2	0.77	89.7±11.6	0.78
No	52	40	36.4±33.9		90.3±10.3	
Liver cirrhosis history						
Yes	83	63.8	32.8±34.9	0.23	87.8±14.0	0.09
No	47	36.2	40.1±32.4		91.2±8.8	
Alpha-fetoprotein (ng/mL) (Mean ± SD)(5)*						
<15	63	50.4	46.7±34.5	<0.01	89.9±9.8	0.65
15–500	42	33.6	32.1±29.5		88.7±14.1	
>500	20	16.0	17.4±28.9		91.5±8.0	

*Number of missing information.

### Preparation of tissue core array

Surgically resected matched pair tissue sections (both adjacent non-tumorous and tumor tissues were originated from the same patient) were collected from 130 CCH HCC patients and all of the pathological sections were reviewed and reported by senior pathologists. Briefly, embedded tissue cores were manually obtained from the tissue blocks by a 10-gauge syringe needle. The cores in demand were placed in a warm cast, which contains melted paraffin wax, and were arranged into an 8 × 12 matrix with an orientated marker from removing the core at the upper-left corner. Each slide contained 96 cores and each patient had two to five cores of their matched pair specimens, depending on the availability of tissue blocks. The positions of each core were recorded on reference sheets to facilitate data acquisition.

### The Cancer Genome Atlas (TCGA) HCC patients and RNA-seq data analysis

RNA sequencing (RNA-seq) data and patients’ clinical and demographic information of HCC patients from TCGA Liver Hepatocellular Carcinoma (LIHC) dataset were downloaded from the TCGA data portal (https://tcga-data.nci.nih.gov/). The results presented here are based on the data generated by the TCGA Research Network: (http://cancergenome.nih.gov/). We selected Illumina HiSeq 2000 RNA Sequencing Version 2 platform for RNA-seq data and we selected 316 HCC tumor tissues and 40 adjacent non-tumorous tissues for further analysis, which are excluded the inconsistent features of HCC patients such as NA pathological stage and zero or NA information in days to death and days to last follow up. The normalized results for mRNA expression, the level 3 expression data from RNA-seq data, of a gene in TCGA Liver Hepatocellular Carcinoma (LIHC) dataset were analyzed by TCGA Research Network through using MapSplice to perform the alignment and RSEM to perform the quantitation. To identify the correlation of selected genes with Hp in either low tumor Hp or high tumor Hp HCC tumor tissues, we calculated the Pearson correlation coefficient and its confidence intervals and *p*-values for each gene by R, version 3.3.1[14].

### Immunohistochemistry staining and assessment

Immunohistochemistry (IHC) staining for Hp was performed by biotin-streptavidin- peroxidase procedures using a Vectastain ABC Kit (Vector Laboratories, Burlingame, CA). Fresh tumor tissues and adjacent non-tumorous liver tissue from HCC patients in tissue core arrays were fixed in formalin and embedded in paraffin. Each sliced serial section is 4 μm and were mounted on glass slides, de-paraffinized with xylene and dehydrated with alcohol according to the manufacturer’s instructions. Antigen retrieval was conducted by heating the slice in a microwave for 2 minutes at 900W then incubated the slides with 0.3% H_2_O_2_ solution in methanol for 30 minutes to block endogenous peroxidase. After rinsing the slides with PBS for three times, the tissues were treated with 20 μg/mL of protease K in the buffer containing 50 mM Tris, 1mM EDTA, 0.5% Triton X-100, pH 8.0 for 15 minutes at room temperature to expose the immunoreactive parts of Hp. Subsequently, the rest of the immunostaining procedures were performed according to the above-mentioned description. Each stained core was scored by Dr. Tsung-Han Teng and Dr. Yan-Ren Lin on the percentage of positively stained (0–100%) and stain intensity (negative, 0; weak, +1; moderate, +2; and strong +3). Total 130 matched pair tissues had two cores available, for which the mean percentage and maximum stain intensity was averaged from two scores. Besides, according to WHO rules and previous references[15, 16], senior pathologists reviewed the stages of cancer cell differentiation of all the tissue sections.

### Statistical analysis

All data analyses, statistical significances and figures, except for Fig 2B and 2C, were performed by R, version 3.3.1[14]. Fig 2B and 2C were plotted by GraphPad Prism version 6.0 (GraphPad Software, La Jolla, CA, USA). Statistical significances between two groups of each sample were analyzed with two-sided Wilcoxon rank-sum test. For multi-groups of samples, the statistical significance was analyzed with one-way ANOVA. The correlation analysis of selected genes with Hp is calculated by the Pearson correlation coefficient. Survival rate analysis was presented in Kaplan-Meier curves and statistical significance is assessed using the log-rank test. The optimal cut point for survival rate analysis is determined by analyzing *p*-values form continuous Hp expression of all the HCC patients, which is based on PrognoScan database[17]. For all statistical analysis, *p*-values of less than 0.05 were considered as statistically significant.

## Results

### Tissue Hp protein expression level is correlated with HCC cancer differentiation in CCH HCC patients

**Fig 1:**
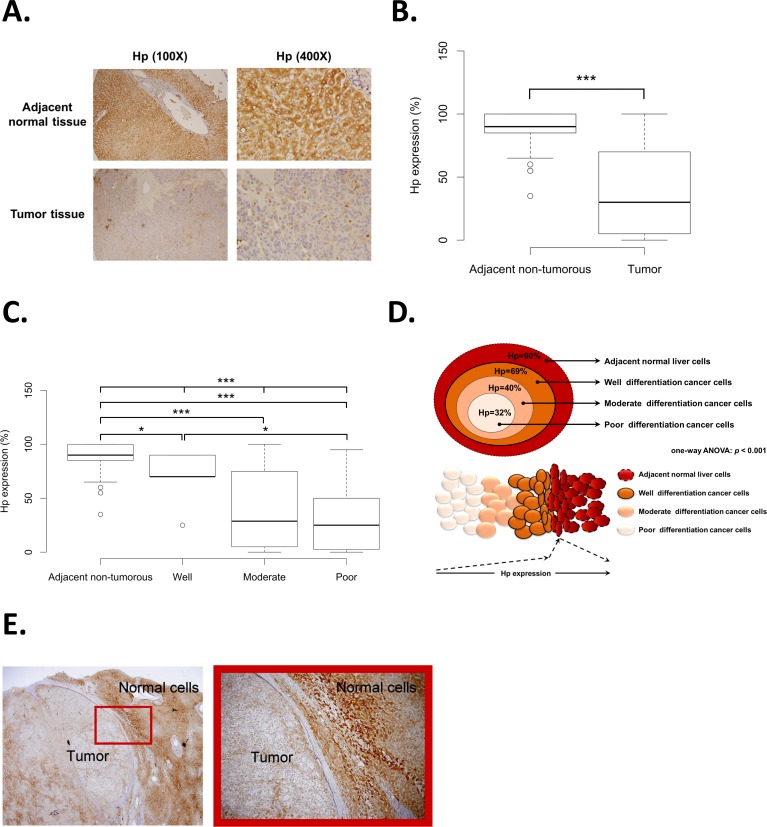
Hp protein expression and cancer differentiation analysis of CCH HCC patients. (A) An example of Hp IHC staining in adjacent non-tumorous tissues and tumor tissues under 100X and 400X magnification. (B) Hp protein expression analysis between matched pair adjacent non-tumorous tissues and tumor tissues of HCC patients from CCH (*p* < 0.001). (C) Boxplot of Hp protein expression in adjacent non-tumorous tissues and three stages HCC cancer differentiation (well, moderate, and poor) (*p* < 0.001). (D) The schematic diagram interpreted the correlation between Hp expression and HCC cancer differentiation (*p* < 0.001). (E) An example of IHC staining for measuring the Hp protein expression at the junction area between adjacent non-tumorous tissue and tumor tissue.

To compare Hp protein expression levels between HCC tumor tissues and adjacent non-tumorous tissues, we analyzed 130 matched pairs of HCC tissue sections, including the pathological stage (stage I, II, III, and IV) with the Hp protein expression level by immunohistochemistry (IHC) staining. The clinicopathological characteristics of 130 HCC patients from CCH cohort are shown in Table 1. Under 100X magnification, the positive Hp staining is presented in adjacent non-tumorous tissue, yet largely absent in tumor tissue (Fig 1A, 100X magnification). Intense Hp staining can be clearly observed in adjacent non-tumorous cells under 400X magnification. In contrast, the tumor portion was presented in negative Hp staining (Fig 1A, 400X magnification). Comparing between matched pair tumor tissues and adjacent non-tumorous tissues of 130 HCC patients, the Hp expression is significantly decreased in tumor tissues than adjacent non-tumorous tissues (*p* < 0.001, Fig 1B). These results indicated that in HCC patients, tissue Hp protein expression levels are reduced in tumor. To further investigate the prognostic value of Hp in HCC cancer progression, we analyzed the correlation between Hp expression levels and HCC cancer cell differentiation. As shown in Fig 1C, among different levels of cancer differentiation subgroups and adjacent non-tumorous tissues, Hp expression was significantly correlated with cell differentiation (one-way ANOVA, *p* < 0.001). Hp protein expression is significantly higher in adjacent non-tumorous cells than other three subgroups of HCC cancer cell differentiation (adjacent non-tumorous vs. well differentiation, *p* < 0.05; adjacent non-tumorous vs. moderate differentiation, *p* < 0.001; adjacent non-tumorous vs. poor differentiation, *p* < 0.001) (Fig 1C). It is worth noting that well-differentiated cancer cells show significantly higher Hp expression than poor-differentiated cancer cells (*p* < 0.05) (Fig 1C). In the schematic diagram of Fig 1D, we demonstrated that HCC cancer cells with better differentiation phenotype show higher Hp expression (one-way ANOVA, *p* < 0.001). The gradient increase of Hp expression is from poor-differentiated cancer cells to well-differentiated cancer cells to the outer adjacent non-tumorous tissue (Fig 1D). By zooming into the junction of tumor part and adjacent non-tumorous part, clear boundary was observed and IHC staining of Hp expression confirmed the results (Fig 1E).

**Fig 2:**
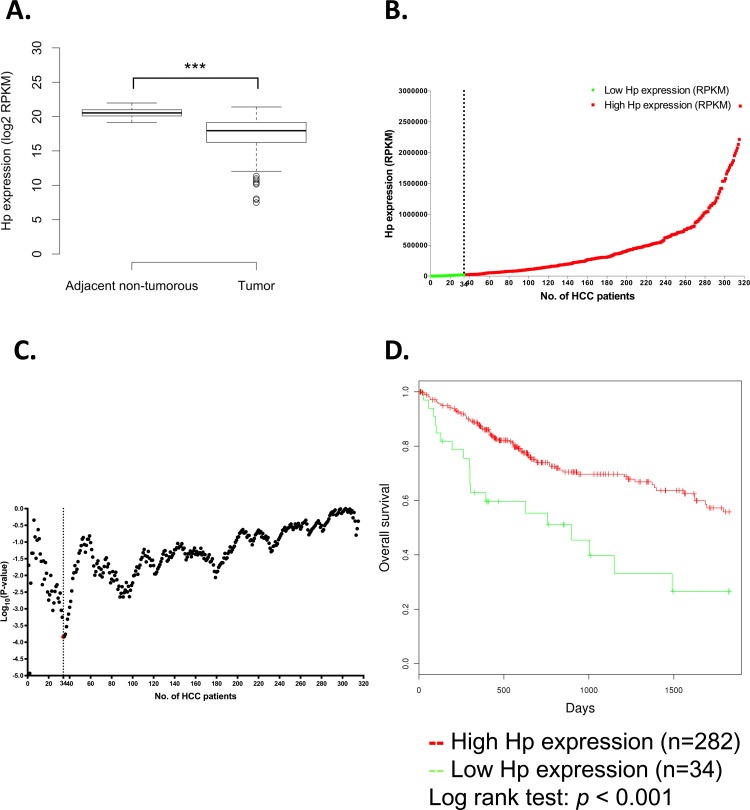
Hp gene expression and five-year overall survival rate analysis in TCGA HCC patients. (A) The boxplot demonstrated Hp gene expression level between adjacent non-tumorous tissues and tumor tissues in TCGA HCC patients (*p* < 0.001). (B) The Hp expression plot showed a total of 316 HCC patients that are ordered by Hp mRNA expression values (RPKM) from the lowest to the highest. The X-axis represents the accumulative number of HCC patients and the Y-axis represents Hp expression value in RPKM. The dashed line shows optimal cut point which dichotomized HCC patients into high (red) and low (green) Hp expression groups. (C) The *p*-value plot displayed the survival significance of each potential cut point of Hp expression measurement. According to each cut point, HCC patients are dichotomized into high and low Hp expression groups, and are calculated by log-rank test for survival significances. The X-axis represents the accumulative number of HCC patients on the order same as the Hp expression plot in panel B and the Y-axis represents raw *p*-values on a log10 scale. The optimal cut point is colored in red and is indicated by dashed line. (D) The Kaplan-Meier plot of HCC survival analysis. HCC patients were dichotomized into low Hp expression group (n = 34) and high Hp expression group (n = 282). The Kaplan-Meier survival curve showed high tumor Hp expression group has increased five-year overall survival rate, comparing with low tumor Hp expression group in TCGA HCC patients (*p* < 0.001).

### Hp expression is correlated with five-year overall survival rate of TCGA HCC patients

To further confirm our clinical results, we next analyzed RNA-seq data of HCC patients in TCGA database. Total 316 HCC patients from TCGA database and the detailed clinical pathological characteristics are displayed in Table 2. As expected, the results showed the similar trend with CCH cohort, which Hp expression is decreased in tumor tissues and also highly expressed in adjacent non-tumorous tissues in TCGA HCC patients (*p* < 0.001, Fig 2A). To evaluate the prognostic value of Hp in HCC patients, we next performed overall survival analysis to all (n = 316) HCC patients from TCGA library. TCGA HCC patients were dichotomized into two groups, low tumor Hp expression (n = 34) and high tumor Hp expression (n = 282), through the optimal cut point, which is determined by calculating *p*-values of continuous Hp expression of TCGA HCC patients (Fig 2B and 2C). As shown in Fig 2B and 2C, HCC patients were ordered by Hp expression from the lowest to the highest (Fig 2B) and were calculated by log-rank test for survival significances (Fig 2C). Surprisingly, the result showed that HCC patients with higher tumor Hp expression (n = 282) were correlated with higher overall survival rate, when comparing to patients with lower Hp expression (n = 34) (log-rank test, *p* < 0.001) (Fig 2D).

**Table 2:** Clinicopathological characteristics of TCGA HCC patients.

	HCC cases (n = 316)		Adjacent non-tumorous tissues (n = 40)	
	No.	%	No.	%
Gender				
Male	216	68.4	22	55
Female	100	31.6	18	45
Age (Mean ± SD) (y/o)	59.6 ± 13		59.9 ± 12.9	
Pathologic_stage				
I	160	50.6	18	45
II	73	23.1	9	22.5
III	80	25.3	12	30
IV	3	1	1	2.5
Pathology_T_stage				
t1	160	50.6	19	47.5
t2	74	23.4	9	22.5
t3	72	22.8	12	30
t4	9	2.9	0	0
NA	1	0.3	0	0
Pathology_N_stage				
n0	231	73.1	30	75
n1	3	1	1	2.5
nx	81	25.6	8	20
NA	1	0.3	1	2.5
Pathology_M_stage				
m0	240	75.9	31	77.5
m1	3	1	1	2.5
mx	73	23.1	8	20
Residual_tumor				
r0	280	88.6	34	85
r1	12	3.8	0	0
r2	1	0.3	1	2.5
rx	16	5.1	3	7.5
NA	7	2.2	2	5
Radiation_therapy				
No	292	92.4	31	77.5
Yes	9	2.9	2	5
NA	15	4.7	4	10
Race				
american indian or alaska native	1	0.3	0	0
asian	145	45.9	5	12.5
black or african american	13	4.1	6	15
white	8	2.5	26	65
NA	149	47.2	3	7.5
Ethnicity				
hispanic or latino	15	4.7	0	0
not hispanic or latino	289	91.5	36	90
NA	12	3.8	4	10

### Hp expression is negatively correlated with poor cancer differentiation markers

To verify our results that lower Hp expression is correlated with poor HCC cancer cell differentiation, we selected several markers (TWIST1, LAMB1, THY1, EZH2, SALL4, and TCF3) that are closely associated with the activation of poor HCC cancer differentiation and analyzed the Pearson correlation coefficient of each selected marker to Hp. Based on the RNA-seq data and previous results of survival analysis, we again divided all stages of HCC patients into two groups, low tumor Hp expression (n = 34) and high tumor Hp expression (n = 282). We then calculated the Pearson correlation coefficient of each marker to Hp in low tumor Hp expression and high tumor Hp expression respectively (Table 3). In low tumor Hp expression group, TWIST1 (r = -0.54, *p* < 0.01), LAMB1 (r = -0.58, *p* < 0.001), and THY1 (r = -0.48, *p* < 0.01) showed negative correlation with Hp. In high tumor Hp expression group, EZH2 (r = -0.53, *p* < 0.001), SALL4 (r = -0.4, *p* < 0.001), TCF3 (r = -0.45, *p* < 0.001) were also negatively correlated with Hp.

**Table 3:** The Pearson correlation coefficient of selected cancer differentiation markers with Hp.

	Low tumor Hp group (n = 34)			High tumor Hp group (n = 282)		
Gene Symbol	Pearson correlation coefficient	95% confidence interval	*p*-value	Pearson correlation coefficient	95% confidence interval	*p*-value
TWIST1	-0.54	-0.74 to -0.24	0.0011	-0.06	-0.18 to 0.06	0.3021
LAMB1	-0.58	-0.77 to -0.30	0.0004	-0.23	-0.35 to -0.12	< 0.0001
THY1	-0.48	-0.70 to -0.16	0.0045	0.08	-0.036 to 0.20	0.1729
EZH2	0.10	-0.24 to 0.43	0.5589	-0.53	-0.61 to -0.44	< 0.0001
SALL4	0.11	-0.23 to 0.43	0.5264	-0.4	-0.49 to -0.30	< 0.0001
TCF3	-0.19	-0.49 to 0.16	0.295	-0.45	-0.54 to -0.35	< 0.0001

## Discussion

The clinical significance of Hp is associated with the progression of diverse malignancies such as autoimmune disorders, diabetes, cardiovascular diseases, and, especially, cancers[18]. Elevated serum Hp expression is often observed in various types of cancer, which is identified as a poor prognostic marker[8, 19, 20]. Previous study showed that serum Hp is highly expressed in HCC patients as compared with liver cirrhosis (LC) patients, suggesting that Hp could be another potential biomarker complementary to alpha-fetoprotein[21]. However, the clinical significance of tumor Hp expression in HCC patients is not yet been revealed. In this study, we first reported the alteration of Hp expression in tumor tissues as compared with adjacent non-tumorous tissues in HCC patients, which can be regarded as a novel prognostic marker for HCC patients.

To the beginning, we examined tissue Hp expression in the two enrolled cohorts, CCH and TCGA HCC patients. As shown in Fig 1A and 1B, we found significantly higher tissue Hp expression in adjacent non-tumorous tissues than in tumor tissues by IHC staining of tissues sections from CCH HCC patients. In addition, as shown in Fig 2A, we validated our clinical observation by analyzing RNA-seq data in TCGA database, which showed the same trend as the tissue sections from CCH HCC patients. Hp expression is often raised in various malignancies. However, our study showed opposite results. We proposed that the reasonable explanation of such result is because of the detected location of Hp expression. Previous studies mostly focused on the serum level of Hp expression, which is in contrast to our observation on tissue levels of Hp expression. Since Hp is a secreted acute phase protein that can also be slightly secreted by other organs[1], except for liver, under inflammation responses, serum Hp expression may gathered from other places instead of originating from the cancer tissues. Furthermore, chronic inflammation in tumor microenvironment reduced the expression level of pro-inflammatory cytokines thereby affecting Hp expression in HCC tumor tissue, since the expressional alteration of Hp expression in hepatocyte in regulated by pro-inflammatory cytokines including IL-6, IL-1β, and TNF-α[22]. In our study, we firstly proved the lower Hp expression in tumor tissues, comparing with adjacent non-tumorous tissues, by directly look into tissue levels of Hp expression through protein and mRNA level.

Given this intriguing results of much higher Hp expression in adjacent non-tumorous tissues than tumor tissues, we next endeavored to assess the potential role of Hp in HCC prognosis. Surprisingly, statistically significant correlation of Hp expression with three stages of cancer differentiation and adjacent non-tumorous cells was discovered (Fig 1C). The gradient increasing Hp expression from poor-differentiated cancer cells to well-differentiated cancer cells to peripheral adjacent non-tumorous cells is illustrated in schematic diagram (Fig 1D). We found that higher tissue Hp expression is correlated with better cancer cell differentiation phenotype. Additionally, the observation of elevated Hp expression in adjacent non-tumorous tissues could be elaborated by the biological stimulation of Hp under inflammation, infections, tissue damages and even stress[23, 24]. Therefore, we proposed that intense Hp expression in adjacent non-tumorous tissues is stimulated by extremely extruding stress from growing tumor (Fig 1D). Furthermore, it might be the reason of elevated serum levels of Hp expression is often detected in diverse cancers in previous reports. IHC staining further showed the intense Hp expression in adjacent non-tumorous tissues and a clear boundary between adjacent non-tumorous tissues and tumor tissues representing well-differentiated cancer cells (Fig 1E). Cancer cell differentiation is closely linked with patients’ prognosis and overall survival rate, since poor differentiated cancer cells are more aggressive than well-differentiated cancer cells, resulting in cancer metastasis and tumor recurrence. As we discovered the statistically significance of higher Hp expression in well-differentiated cancer cells, we next proceeded to used tumor Hp expression as the factor in analyzing five-year overall survival rate to assess whether Hp could serve as a prognostic marker in HCC patients. Through analyzing the *p*-value of each HCC patients, which is ordered by Hp expression, the result provided the optimal cut point for survival analysis and displayed the potential of Hp as a prognostic value in HCC patients (Fig 2B and 2C). In agreement with previous results, Kaplan-Meier overall survival analysis of HCC patients proved that HCC patients with high tumor Hp expression result in improved five-year overall survival rate. By contrast, HCC patients with low tumor Hp expression showed a shorter life expectancy (Fig 2D). Based on these evidences, we revealed that higher Hp expression in tumor tissues is not only correlated with better cancer cell maturation, but also correlated with increasing five-year overall survival rate. We thus demonstrated that the higher tissue Hp expression is a potentially prognostic marker with the better outcome for HCC patients.

Lastly, not merely conclude by the clinical observation and survival analysis, we further attempted to explore the groups of genes, which are related to promoting poor cancer cell differentiation, is correlated with Hp. The six selected genes (TWIST1, LAMB1, THY1, EZH2, SALL4, and TCF3) are involved in cell differentiation, HCC cancer stem cells (CSCs) markers, and CSCs involved pathways. The negative Pearson correlation coefficient demonstrated negative correlations of these selected genes with Hp in either low tumor Hp expression group (n = 34) or high tumor Hp expression group (n = 282) (Table 3). TWIST1 is one of the key transcription factor in activating epithelial-mesenchymal transition (EMT), which is a crucial process for HCC cell de-differentiation[25]. According to previous study, Hp expression is associated with EMT in buccal cancer patients[26]. Another selected marker for cell differentiation is LAMB1, which is in the family of extracellular matrix glycoprotein and is implicated with cell differentiation. Up-regulation of LAMB1 showed poor differentiation and aggressive cancer phenotypes such as migration and invasion[27, 28]. THY1(CD90) is one of the representative HCC Cancer Stem Cells (CSCs) markers. CSCs represents undifferentiated cells that bear stem-like properties and differentiation ability, which contributes to poor prognosis and recurrence[29]. Thus, the negative correlation of TWIST1 (r = -0.54, *p* < 0.01), LAMB1 (r = -0.58, *p* < 0.001), and THY1 (r = -0.48, *p* < 0.01) in low tumor Hp expression groups could be a signature of poor cancer differentiation also the prognostic role of Hp in HCC cancer cell differentiation. On the other hand, negative correlation of EZH2 (r = -0.53, *p* < 0.001), SALL4 (r = -0.4, *p* < 0.001), TCF3 (r = -0.45, *p* < 0.001) with Hp in high tumor Hp expression group could also be an indication of relative well cancer differentiation. EZH2 and SALL4 are two critical genes in regulating CSC properties. Previous study indicated that EZH2 closely regulates differentiation of hepatic stem/progenitor cells[30, 31]. SALL4 is also reported in increasing stem cell properties and causing poor prognosis[31, 32]. Last but not least, TCF3 is one of the components in Wnt/β-catenin signaling pathway, which plays important role in activating liver cancer stem cells[33]. Taken together, the correlation of these selected genes (TWIST1, LAMB1, THY1, EZH2, SALL4, and TCF3) not only as a signature of poor cancer differentiation in low tumor Hp expression group and relative well cancer differentiation in high tumor Hp expression group, but also demonstrates the correlation of Hp expression and cancer differentiation.

To conclude, our data suggested that Hp expression could serve as a novel prognostic marker for HCC patients, which is correlated with cancer cell differentiation and five-year overall survival rate. Our findings contribute in several clinical significances, suggesting Hp can potentially predict HCC prognosis by cancer cell differentiation and then provide information for adequate clinical managements.
